# Effect of Sodium Chloride on the Modulus and Fatigue Life of Bituminous Mixtures

**DOI:** 10.3390/ma13092126

**Published:** 2020-05-03

**Authors:** Luis Juli-Gándara, Ángel Vega-Zamanillo, Miguel Ángel Calzada-Pérez, Evelio Teijón-López-Zuazo

**Affiliations:** 1 GCS Research Group, Civil Engineering School, Universidad de Cantabria, 39005 Santander, Spain; vegaa@unican.es (Á.V.-Z.); calzadam@unican.es (M.Á.C.-P.); 2 Construction and Agronomy Department, University of Salamanca, 49022 Zamora, Spain; eteijon@usal.es

**Keywords:** salt, NaCl, bituminous mixture, resilient modulus, dynamic modulus, fatigue life, winter road

## Abstract

Bituminous mixtures are not perfectly elastic materials, so their viscoelastic properties play a decisive role in knowing their behavior. This research aims to find out this behavior through the values of the resilient modulus, the dynamic modulus, and the fatigue life for asphalt concrete and porous mixtures when they are influenced by the presence of salt (NaCl, sodium chloride). The aforementioned influence of salt has been evaluated by utilizing three different methods: submerging specimens of bituminous mixture in salt water; introducing salt into the specimens as if it was aggregate and immersing the aggregate in salt water; and drying it and then manufacturing the bituminous mixture with it. As the results indicate, the mixtures submerged in salt water do not show large differences in comparison to the reference mixtures for hot mix asphalt and porous mixtures. However, for hot mix asphalt in which salt has been added as aggregate, the resilient modulus is greater than in the reference mixture. For the bituminous mixtures in which an aggregate saturated in salt water has been used for its manufacture, the results of the resilient modulus test, dynamic modulus test, and fatigue life test are lower than in the reference mixture, especially when the specimens are submerged.

## 1. Introduction 

Sodium chloride (NaCl) is one of the most commonly used materials in civil engineering to combat the formation of ice on roads. To prevent the formation of this ice, salt can be spread on the surface of the bituminous mixture or included in the mixture as an additive, forming part of the aggregate. Salt is used as a deicing agent, stemming from its adaptability, intrinsic properties, and above all, the fact that it is cheaper than the rest of the deicing agents [1,2]. According to García [2], NaCl is more than eight times cheaper than calcium chloride (CaCl_2_), the second cheapest deicing agent. In addition, García noted that salt can be used effectively from 0 °C to −5 °C, and even down to −21 °C in combination with CaCl_2_. This is a typical Spanish winter temperature range.

Nevertheless, NaCl has a negative environmental impact which has been exhaustively studied. The spreading of NaCl over the pavement generates an alteration in the amount of heavy metals (chromium, lead, or molybdenum) [3,4,5,6] and an increase in chlorides [7,8] in the immediate areas surrounding the roads. In order to minimize this negative impact, Ikiz et al. [9] designed a decision tree and Trenouth et al. [10] implemented a computer application to know with precision when salt should be applied in order to reduce the amount of NaCl used on winter roads. Following this trend of reducing the amount of NaCl spread, Klein-Paste et al. [11] noted that using 60% of the salt currently applied is sufficient to weaken the layers of ice that are formed on road surfaces.

The mechanical behavior of bituminous mixtures that have been in contact with NaCl in comparison with other deicing agents was also researched. These investigations noted that NaCl does not generate a polished road surface in comparison with sand or quartz dust [12]. Furthermore, the value of ITS (Indirect Tensile Strength) after 50 freeze–thaw cycles for mixtures exposed to NaCl was greater than other deicing agents, even better than the reference series [13].

Some researchers [14,15] proposed the use of additives compounded principally by salt (sodium chloride or calcium chloride) and integrated as aggregate in the bituminous mixture in order to prevent the formation of ice on the roads. Using an additive similar to these, Liu et al. [16] conducted research to determine the ITS and Indirect Tensile Strength Ratio (ITSR) of a bituminous mixture with different fineness of an antifreeze additive and the results show a 7% increase in the values of ITSR using the finest additive compared with the reference.

The effect salt has on bituminous mixtures may not only be due to winter roads. Feng et al. [17] researched three types of mixtures with different compositions (AM, OGFC, and AC) subjected to freeze–thaw cycles while submerged in seawater. They concluded that the values of ITSR are lower for all the mixtures compared with the reference series, but the AC has values closer to the reference than the other two mixtures. Obika et al. [18] noted that solubility, salt crystallization, and crystal pressures are the determining factors in the damage mechanism for the bituminous mixture in hot climates.

Juli-Gándara et al. [19] collected the mechanical performance of an asphalt concrete and a porous asphalt (with two different binders) influenced by different interactions with salt. This influence of NaCl was examined by submerging specimens in salt water, adding NaCl as an additive into the bituminous mixture and immersing the aggregate in salt water before using it to manufacture the mixture. Their results show that the values of the ITS, ITSR, Wheel Tracking Test, and Cantabro Loss Particle Test are slightly affected by NaCl when the bituminous mixture is submerged in salt water. However, the mixtures that have salt as additive—even more in the case of the porous asphalt—have lower values of the ITS and Cantabro Loss Particle Test due to the fact that NaCl is softer than the aggregate. Furthermore, the aggregate–binder interface and the adhesiveness for bituminous mixtures in which its aggregate was immersed in salt water before manufacture are greatly affected.

However, the behavior of bituminous mixtures are not utterly defined without knowing its elastic and viscoelastic properties [20,21]. Tino [22] researched the effect of external agents, including salt as a deicing agent, on the viscoelastic behavior of bituminous mixtures. He concluded that the most negative effect for them is the combination of climate factors (principally, temperature and humidity), aggravated by the action of salt as a deicing agent. Vega-Zamanillo et al. [23] studied the impact of temperature changes and freeze–thaw cycles on the behavior of bituminous mixtures, including viscoelastic properties, submerged in salt water. They indicated that the most damaging process takes place when the mixture remains in contact with frozen water. They also noted that the specimens submerged in salt water maintain their mechanical properties better than those submerged in distilled water.

This research will attempt to define the elastic and viscoelastic properties through the values of the resilient modulus, dynamic modulus, and life fatigue of two bituminous mixtures—an asphalt concrete and a porous asphalt—when influenced by the presence of NaCl. This influence has been evaluated by three different methods: submerging specimens of bituminous mixture in salt water; introducing salt into the specimens as aggregate and immersing the aggregate in salt water; and drying it and manufacturing the bituminous mixture with it.

## 2. Materials and Methods

### 2.1. Materials

#### 2.1.1. Binder

In this research, a bitumen commonly used in Spain (B 50/70) has been utilized. The characteristic values of this bitumen are as follows:

Frass Breaking Point (UNE-EN 12593:2015) [24] −9 °C.
Softening Point (UNE-EN 1427:2015) [25] 47.2 °C.
Penetration (UNE-EN 1426:2015) [26] (25 °C; 100 g, 5 s) 65.0 (0.1 mm).

#### 2.1.2. Aggregate

All the specimens have been manufactured using an ophite with the following properties:

Specific Weight (UNE-EN 1097-6:2014) [27] 2.921 g/cm^3^.
Water Absorption (UNE-EN 1097-6:2014) [27] 1.0%.
Los Angeles Abrasion Test (UNE-EN 1097-2:2010) [28] 16.0%.
Flakiness Index (UNE-EN 933-3:2012) [29] 9.0%.
Limestone (specific weight 2.753 g/cm^3^) has been used as mineral powder.

#### 2.1.3. Bituminous Mixture

**Figure 1:**
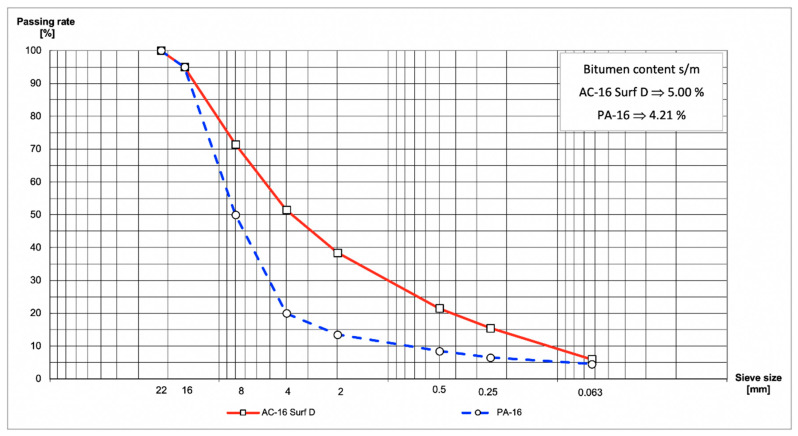
Particle size and asphalt content of bituminous mixture.

The two mixtures selected to compose this research were an AC-16 Surf B 50/70 D with a binder content of 5.00% and a PA–16 with a binder content of 4.21% (composition in Figure 1). Both are commonly used in Spain and appear in articles 542 and 543 of the Spanish Standard PG–3 [30]. AC–16 Surf B 50/70 D is one of the most common mixtures used on high mountain roads, and the kind most likely to have winter road problems. PA–16 is usually used on high-capacity highways, including highways around marine environments. All of the specimens have been manufactured in a laboratory using a mixer with planetary rotation and vertical shaft.

The variation in density and air void content (UNE-EN 12697–34:2013) [31] of all the different specimens is shown below:

- Asphalt concrete:
  Density: 2.45–2.51 g/cm^3^.
  Air void content: 6.0–8.4%.
- Porous asphalt:
  Density: 2.17–2.22 g/cm^3^.
  Air void content: 16.7–19.8%.

#### 2.1.4. NaCl and Seawater

**Figure 2:**
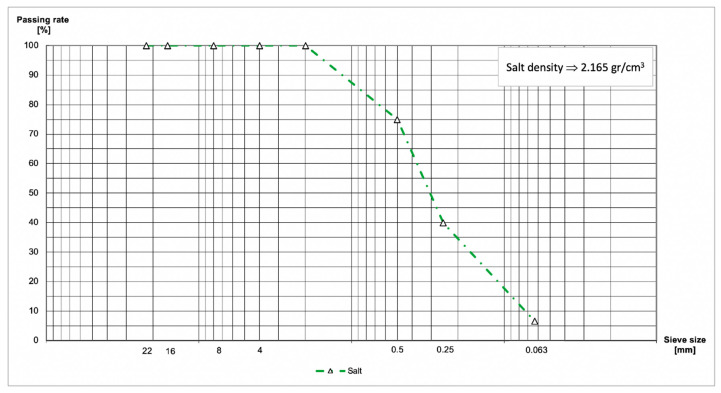
Passing rate and density of salt.

NaCl is the salt used in this research. Passing rate and density are shown in Figure 2.

For one of the cases of specimens submerged in salt water, seawater from the Bay of Santander (Spain) was used. This seawater was provided by the Maritime Museum of the Cantabrian Sea with a salt concentration of 3.5% totally dissolved.

### 2.2. Methodology

Three different salt treatments were analyzed. Series A is for references.

#### 2.2.1. Bituminous Mixture Immersed in Salt Water

This treatment is similar to the conditions of a bituminous mixture subjected to the spread of brine on a winter road or a road that is in close proximity to seawater. The specimens are immersed in three different saltwater concentrations:

Series B1, 3.5% (seawater).
Series B2, 5.0% amount of salt by weight of distilled water.
Series B3, 10.0% amount of salt by weight of distilled water.

The time and temperature that the specimens remain submerged in salt water depend on the particular test.

#### 2.2.2. NaCl Introduced as Aggregate into the Bituminous Mixture

By adding NaCl as aggregate (not a replacement of aggregate) into the specimens, this research attempts to simulate the use of an additive that is utilized to prevent the formation of ice on roads. Two different amounts of salt by weight of aggregate were used:

Series C1, 5%.
Series C2, 10%.

Salt was added at the same time as the aggregate when the bituminous mixture was mixed.

#### 2.2.3. Bituminous Mixture Manufactured with an Aggregate Previously Saturated in Salt Water

Through this treatment, the research attempts to find out how the aggregate–binder interface is affected by the action of salt. The method that this investigation follows in order to saturate the aggregate with salt is to submerge all of the aggregate in salt water for 72 h at 20 °C (enough time to be totally saturated). Afterwards, the aggregate remained in the same container and was dried for 24 h at 60 °C. Lastly, the aggregate was transferred to a metal container, in which it reached the required mixing temperature. 

The aggregate was submerged using two different amounts of salt by weight in distilled water:

Series D1, 2.0%,
Series D2, 3.5%.

### 2.3. Tests

#### 2.3.1. Resilient Modulus Test

**Figure 3:**
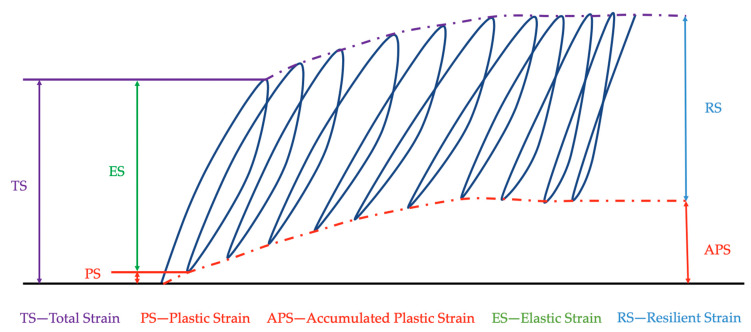
Resilient modulus.

One of the most important features for typifying a material in civil engineering is its elastic properties, even in this case when the bituminous mixture has a viscous component. A bituminous mixture is a material that is subjected to dynamic load–unload cycles. The material strain under these cycles is divided into two parts: plastic strain; and elastic or recoverable strain. When the number of cycles increases, the accumulated plastic strain remains constant. At this moment, all of the strain is recoverable, and the behavior of the mixture is called resilient (Figure 3). This characteristic is also important in order to know its mechanical behavior. The test was conducted in accordance with the standard UNE-EN 12697-26: 2012 “Annex C” [32], applying indirect tension to cylindrical specimens. The dimensions of these cylindrical specimens are shown in the standard. Eight dry and another eight wet specimens were tested for each series. The wet specimens remained submerged in a water bath at 40 °C for three days before drying at 20 °C—the temperature at which the test is carried out.

#### 2.3.2. Dynamic Modulus Test

As previously mentioned, bituminous mixture is not a perfectly elastic material, it has a viscous component. Due to this fact, the values of the dynamic modulus are of primary importance. This test follows the directive of the standard UNE-EN 12697-26: 2012 “Annex B” four-point bending test [32]. The testing temperature was 20 °C. The dynamic modulus test was carried out for one wet series of salt treatments, C and D of PA-16, because these series of porous asphalt are most susceptible to being affected by salt [19]. The wet specimens follow the same procedure as the wet specimens of resilient modulus. The dimensions of the beam used in this test appear in the standard. Eight specimens were tested from each selected series.

#### 2.3.3. Fatigue Test

The fatigue test furnishes an idea of the durability of a bituminous mixture through cycles of dynamic load and unload. UNE-EN 12697-24: 2012 “Annex D” four-point bending test [33] provides the method to carry out the test. Test temperature, series, dimensions, number of specimens evaluated, and the procedure for wet specimens are also the same as in the dynamic modulus test. 

## 3. Results

### 3.1. Resilient Modulus Test

The results generated in this test (Table 1 and Table 2) provide an idea that salt has a scant impact on the resilient modulus when the action of the salt takes place after the manufacture of the bituminous mixture (B-Series), however, for the series in which the salt treatment takes place before the manufacture (C and D series), the modulus is altered.

The specimens of B-Series, submerged in any saltwater concentration, for both types of mixtures as well as for dry and wet specimens, have values of resilient modulus similar to the reference series. The modulus has more variability only for wet specimens of porous asphalt.

Dry specimens of C-Series, for asphalt concrete and porous asphalt, reach values slightly higher of resilient modulus than those of A-Series. This may be due to the fact that the addition of salt forces the binder to blend with more aggregate, so the percentage of bitumen content is reduced. For wet specimens however, the two mixtures display different behavior. The values of resilient modulus of wet asphalt concrete specimens increase between 4% and 26%, reaching values higher than their analogous dry specimens (C2) for the same reason as in the dry specimens. Nevertheless, porous asphalt has a completely different behavior. Even though it is true that in this mixture the percentage of bitumen content is also reduced, there is another more decisive aspect; porous asphalt is more susceptible to the action of water, and this fact involves a decrease in the values of the modulus between 10% and 21%.

D-Series shows results of resilient modulus much lower than those of the reference series. On account of the fact that salt impregnates the aggregate, the aggregate–binder interface is critically damaged, losing all the adhesiveness; likewise, this is even stronger in porous asphalt when the mixture is submerged in water with the highest quantity of salt.

### 3.2. Dynamic Modulus Test

**Figure 4:**
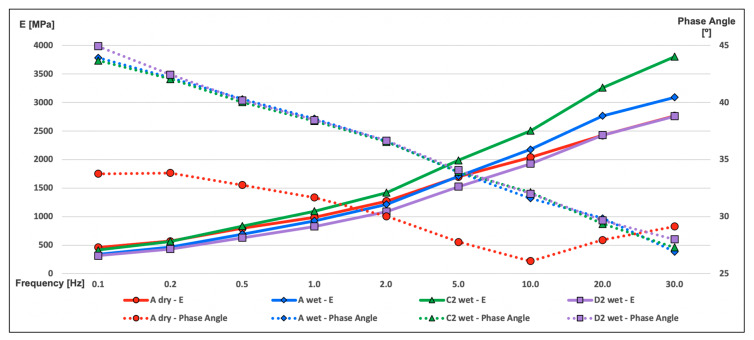
Dynamic modulus test.

Figure 4 shows that wet reference specimens have a phase angle higher than dry reference specimens. So, submerging the mixture in water is the first factor in regard to lose elasticity.

Furthermore, the specimens in which salt was added as aggregate have values of the modulus higher than those of the reference series, reaching values even higher than the dry specimens of A-Series for frequencies beyond 0.5 Hz. In addition to the greater phase angle, this fact indicates that C-Series is more viscous than A-Series.

Just as the results of the resilient modulus, D-Series in this test has the lowest values of dynamic modulus for all of the frequency ranges. Parallel to this, the phase angle has higher values than the rest of series. These results corroborate the hypothesis that the aggregate–binder interface is damaged by this salt treatment.

The goodness-of-fit statistic is shown in Table 3 and the analysis of variance appears in Table 4.

### 3.3. Fatigue Test

**Figure 5:**
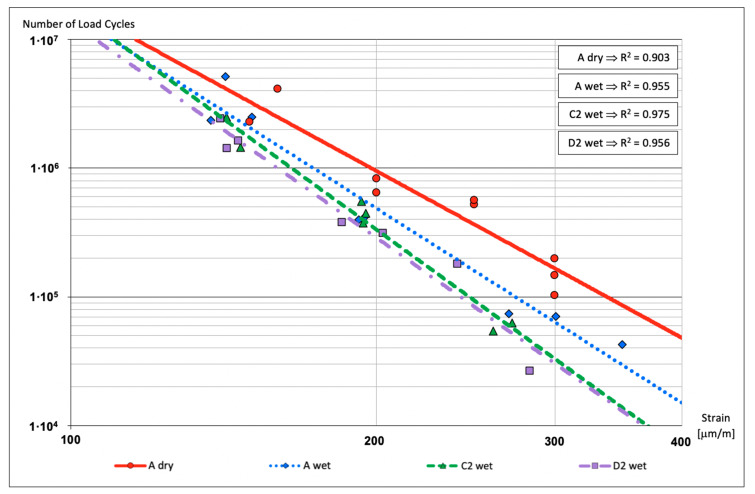
Fatigue test.

The lines that connect the number of load cycles with the strain of the specimens, the fatigue lines (Figure 5), show that the dry reference series obtains a greater number of load cycles than the rest of series for all of the strain ranges. This result is due to the fact that the A dry series does not undergo any type of damage process. Nonetheless, in the same figure it could be observed that the rest of the analyzed series are very close, raising the A wet series a few more load cycles than C2 wet and D2 wet that are almost in the same line. Therefore, even though submerging specimens in water is the main damage process for the bituminous mixture, this process in combination with any salt treatment reduces the number of load cycles even more.

The statistical results are shown in Table 5 and Table 6, goodness-of-fit statistic and the analysis of variance, respectively.

## 4. Conclusions

Submerging porous asphalt in water is one of the main damage processes that causes loss of elasticity and durability. However, if salt is added as aggregate in porous asphalt, when the bituminous mixture is submerged, the viscosity increases for all frequencies and the durability decreases even more.

Adding salt as aggregate into the asphalt concrete increments its resilient modulus, even more for specimens that remain submerged, reaching, for the mixture with a 10% of salt added, a value 26% higher than the reference series. Furthermore, in dynamic modulus test, these specimens have values of the modulus higher than those of the reference series, reaching values even higher than the dry reference specimens for frequencies beyond 0.5 Hz.

Immersing specimens in salt water has a scant effect on its resilient modulus compared with submerging the mixture in distilled water. The action of the salt in this case takes place after the manufacture of the bituminous mixture and NaCl cannot damaged the aggregate–binder interface. 

The most harmful effect that NaCl produces in the bituminous mixture is when it affects the aggregate–binder interface, losing all the adhesiveness. This is even more when the mixture is submerged in water. Consequently, the elastic and viscoelastic properties of the bituminous mixture are critically altered. For this reason, the treatment that introduces more damage to bituminous mixture is to use an aggregate previously saturated in salt water in the manufacture of the mixture.

## Figures and Tables

**Table 1:** Resilient modulus.

	Series	Resilient Modulus (MPa)			
		AC-16 Surf D		PA-16	
		Dry	Wet	Dry	Wet
Reference	A	5146	4710	1875	1864
Specimens immersed in salt water	B1	5146	4583	1875	1670
	B2	5146	4544	1875	1479
	B3	5146	4603	1875	2246
NaCl introduced as aggregate	C1	5215	4927	1936	1670
	C2	5470	5936	2289	1470
Aggregate previously saturated in salt water	D1	3873	4378	1813	1712
	D2	3258	3649	1774	569

**Table 2:** Resilient modulus. Standard deviation.

	Series	Standard Deviation (MPa)			
		AC-16 Surf D		PA-16	
		Dry	Wet	Dry	Wet
Reference	A	378	320	208	168
Specimens immersed in salt water	B1	378	312	208	203
	B2	378	342	208	184
	B3	378	350	208	192
NaCl introduced as aggregate	C1	278	301	236	211
	C2	399	410	315	268
Aggregate previously saturated in salt water	D1	287	354	305	242
	D2	354	298	289	290

**Table 3:** Goodness-of-fit statistic. Dynamic modulus.

R²	0.971
Adjusted R²	0.967

**Table 4:** Analysis of variance. Dynamic modulus.

Source	DF	Sum of Squares	Mean Squares	F	Pr > f
Model	5	31,629,381.51	6,325,876.30	203.63	<0.0001
Error	30	931,953.49	31,065.12	-	-
Corrected Total	35	32,561,335.00	-	-	-

Computed against model Y = Mean (Y)

**Table 5:** Goodness-of-fit statistic. ${\mathrm{Log}}_{10}$ (Number of load cycles) - ${\mathrm{Log}}_{10}$ (Strain). Fatigue.

R²	0.972
Adjusted R²	0.967

**Table 6:** Analysis of variance. ${\mathrm{Log}}_{10}$ (Number of load cycles) - ${\mathrm{Log}}_{10}$ (Strain). Fatigue.

Source	DF	Sum of Squares	Mean Squares	F	Pr > f
Model	4	11.29	2.82	222.16	< 0.0001
Error	27	0.33	0.01	-	-
Corrected Total	31	11.62	-	-	-

Computed against model Y = Mean (Y)
